# Office Visitors

**Published:** 1907-05

**Authors:** 


### OFFICE VISITORS.

We had met, at a convention, an old school mate, we both had spent our boyhood in the city where the convention convened, the dialect of which city was of such peculiarity that it is markedly and unmistakably different from anywhere else in the land. During a trolley ride to a suburban park, to which we resorted for a long talk of the old times and since, we remarked that many years absence from the historic city had completely eliminated from our tongues the distinctive accent of the place. We individually went so far as to assert that having practised at a resort visited by the people of every section we had become an expert in locating individuals by their accent. On a seat in front of us sat two men who afterwards being in our company one of them said to us: "We were much amused at your conversation which we overheard and we would like to ask if you could locate us by our speech." At that period our state was being visited here and there by Mormon missionaries with some of whom we had previously come in contact, we put this and that together and said, "I think you are from the far west." "How strange" said they "that is correct, now from what state?"

"I should judge you are from Utah," we replied. "Well now did you ever see or know any of us before?" We said "No." They thought it very strange, but were more so when we expressed the opinion that they were elders of the Church of Latter Day Saints. Our friend and ourselves took advantage of the acquaintance thus made and plied them with many questions, as to their faith and their mission, treating them the while, with respect and politeness. We parted cordially. A short time after our return home two gentlemen were ushered in our office and bade us "good morning," with the accent of our companions du voyage. They informed us that on the recommendation of the latter, whom they were in touch with, they had called to see us and have us give them professional services. They seemed to be impressed with the idea that we were a favorable subject for proselyting and we humored the notion to the extent that we were very desirous of light on the subject of their faith, at the same time not committing ourselves to them. We gained their confidence and regard, and they supplied us with numerous tracts and a Book of Mormon. We soon learned that quite a number of them were in the city and in course of a few weeks we had made the acquaintance of all and had a very remunerative run of practice among them. Well, we took those tracts and that Mormon bible home, and during our spare moments would read them. It was not long before the household felt called on to ask questions. "What are you wasting your time reading that unreasonable parody on religion for?" asked a devoted Baptist brother. "Father said the literary daughter, "their writings are a very poor imitation of the holy scripture and they are blasphemous." But it was when having finished the tracts and had gotten pretty well into Joseph Smiths bible and had intentionally on purpose, ejaculated sundry, "well that sounds reasonable." "Polygamy seems to have been a sanctified revelation and practice" and "I wonder if Utah is a good climate and if they don't have their plural wives in secret?" that the wife of our bosom felt called on to interfere and she did with a fullness of expression that was "a plenty," to use slang. "Now you will please understand that if you have lost your senses or have been really influenced by those Mormons and desire to go to Utah, you can "went," but by yourself and that there are other states besides this that can grant me a divorce." The time had come for explanations and we made them quick. We are happy as ever now, but that bible and those tracts have disappeared and we dare not make inquires of their whereabouts.

